# Heterologous vaccination against human tuberculosis modulates antigen-specific CD4^+^ T-cell function

**DOI:** 10.1002/eji.201343454

**Published:** 2013-07-08

**Authors:** One B Dintwe, Cheryl L Day, Erica Smit, Elisa Nemes, Clive Gray, Michele Tameris, Helen McShane, Hassan Mahomed, Willem A Hanekom, Thomas J Scriba

**Affiliations:** 1 South African Tuberculosis Vaccine Initiative and School of Child and Adolescent Health, Institute of Infectious Disease and Molecular Medicine, University of Cape TownCape Town, South Africa; 2 Department of Global Health, Rollins School of Public Health, Emory UniversityAtlanta, GA, USA; 3 Emory Vaccine Center, Emory UniversityAtlanta, GA, USA; 4 Division of Immunology, Institute of Infectious Disease and Molecular Medicine, University of Cape TownCape Town, South Africa; 5 Centre for Clinical Vaccinology and Tropical Medicine and The Jenner Institute Laboratories, Nuffield Department of Medicine, Oxford UniversityOxford, United Kingdom

**Keywords:** HLA class II tetramer, MVA85A, Proliferation, T cells, Vaccine

## Abstract

Heterologous prime-boost strategies hold promise for vaccination against tuberculosis. However, the T-cell characteristics required for protection are not known. We proposed that boost vaccines should induce long-lived functional and phenotypic changes to T cells primed by Bacille Calmette Guerin (BCG) and/or natural exposure to mycobacteria. We characterized changes among specific CD4^+^ T cells after vaccination with the MVA85A vaccine in adults, adolescents, and children. CD4^+^ T cells identified with Ag85A peptide-bearing HLA class II tetramers were characterized by flow cytometry. We also measured proliferative potential and cytokine expression of Ag85A-specific CD4^+^ T cells. During the effector phase, MVA85A-induced specific CD4^+^ T cells coexpressed IFN-γ and IL-2, skin homing integrins, and the activation marker CD38. This was followed by contraction and a transition to predominantly IL-2-expressing, CD45RA^−^CCR7^+^CD27^+^ or CD45RA^+^CCR7^+^CD27^+^ specific CD4^+^ T cells. These surface phenotypes were similar to Ag85A-specific T cells prior to MVA85A. However, functional differences were observed postvaccination: specific proliferative capacity was markedly higher after 6–12 months than before vaccination. Our data suggest that MVA85A vaccination may modulate Ag85A-specific CD4^+^ T-cell function, resulting in greater recall potential. Importantly, surface phenotypes commonly used as proxies for memory T-cell function did not associate with functional effects of vaccination.

## Introduction

After clean water, vaccination is the most effective global public health intervention [1]. While protection by most currently licensed vaccines correlates with levels of induced antibodies, protection against pathogens such as HIV-1 and *Mycobacterium tuberculosis* (*M. tb*) is thought to rely, at least in part, on specific T-cell responses [2, 3]. Heterologous prime-boost regimens, involving priming with either BCG or an improved live mycobacterial vaccine, followed by an adjuvanted subunit or viral vectored boost, may constitute the most promising vaccination strategy against tuberculosis (TB) [4–6].

It is currently not known exactly which T-cell response vaccines should induce for increased protection against TB disease [2, 3]. In phases I and II clinical trials of new TB vaccines, the frequencies of vaccine-induced antigen-specific T helper type 1 (Th1) cytokine-expressing CD4^+^ and/or CD8^+^ T cells are usually quantified with the premise that vaccination-induced responses should be higher than the prevaccination response [7]. The pattern of effector cytokine expression by specific T cells is also commonly measured [7–9]. However, we recently showed that a Th1 response-inducing vaccination strategy in infants, which involves a BCG prime at birth and a boost with the novel poxvirus-vectored TB vaccine candidate, MVA85A, showed no evidence of efficacy against TB disease or *M. tb* infection [10]. These results suggest that features other than frequencies and cytokine-expression patterns of induced T cells should be explored as correlates of vaccine-induced immunity. For example, it is thought that the capacity to expand after T cells reencounter antigen is an important function that may be measured in vaccine trials [11].

The success of heterologous boost vaccines may depend on the modulation of the existing mycobacteria-specific T-cell repertoire to possess more “favorable” functional characteristics, rather than inducing de novo T-cell responses. In TB endemic countries, CD4^+^ T cells specific for conserved immunodominant antigens such as Ag85A are detectable in most individuals beyond infancy [12]. These cells could have been induced by BCG vaccination and/or exposure to environmental mycobacteria and/or *M. tb* or even cross-reactive bacteria [8, 12, 13]. We propose two minimum criteria for a potentially successful heterologous vaccination strategy: (1) the boost vaccine should modify or reprogram the T-cell response to display different functional and/or phenotypic characteristics to the prevaccination response; (2) the induced T-cell response should be long lived.

In the present study, we comprehensively characterized mycobacteria-specific CD4^+^ T cells before and after vaccination with MVA85A. We showed that changes in commonly measured phenotypic markers of MVA85A-induced CD4^+^ T cells were either short-lived (acute effector response) or equivalent to the prevaccination Ag85A-specific CD4^+^ T-cell response. However, MVA85A vaccination modulated the proliferative capacity of Ag85A-specific CD4^+^ T cells, which was markedly higher 6–12 months after MVA85A vaccination, than before vaccination.

## Results

### Ex vivo detection of Ag85A-specific CD4^+^ T cells by DR3-Ag85A HLA class II tetramer staining

**Figure 1:**
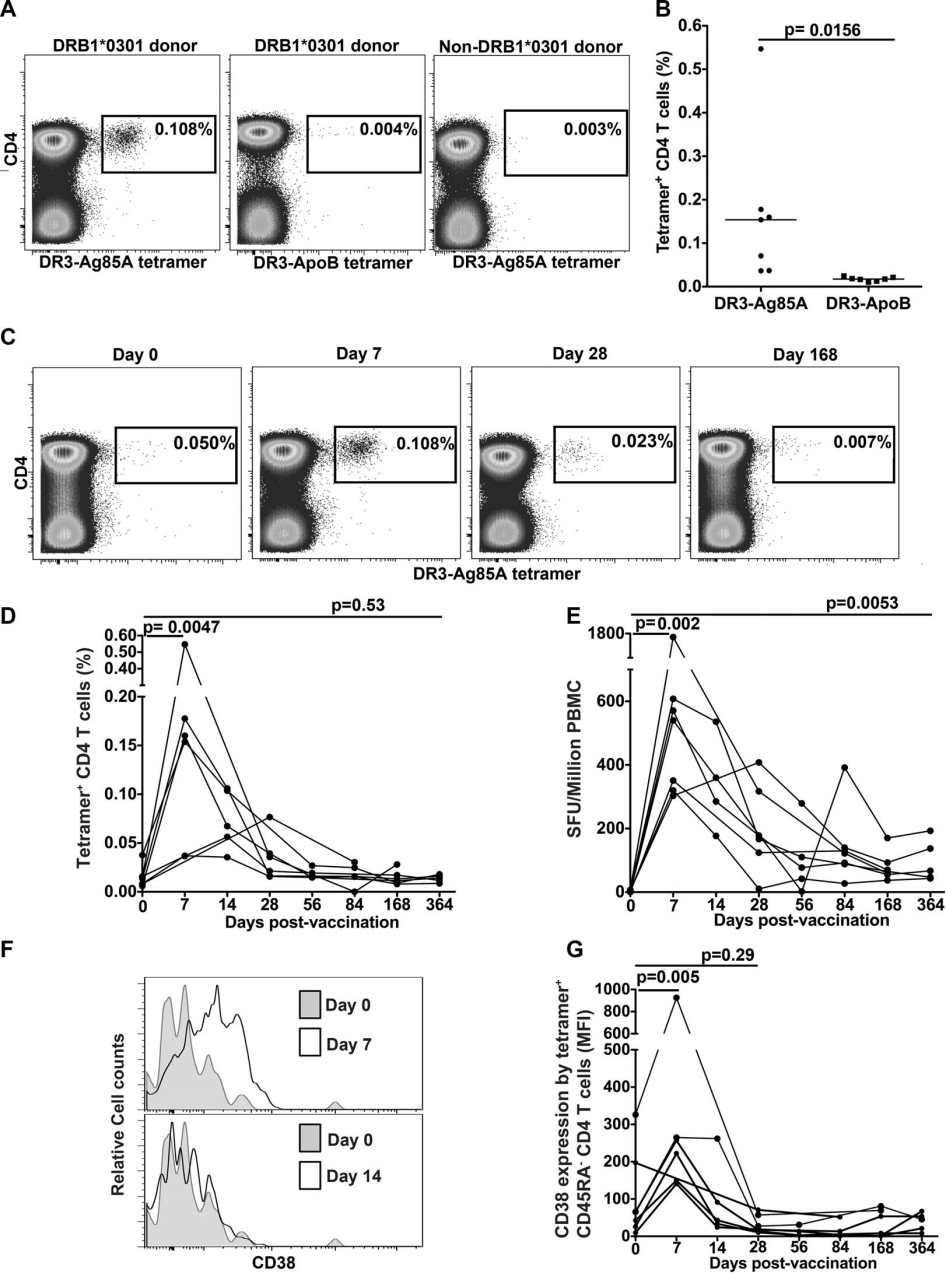
Direct ex vivo detection of mycobacterial Ag85A-specific CD4^+^ T cells by HLA class II tetramer staining. PBMCs from MVA85A-vaccinated individuals were stained with the DR3-Ag85A tetramer or the DR3-ApoB control tetramer. Flow cytometry plots show data gated on CD14^−^, CD19^−^, live (ViViD^−^), CD3^+^ lymphocytes. The gating strategy is shown in Supporting Information Fig. 1A. (A) HLA class II tetramer staining of PBMCs 7 days after MVA85A vaccination, from a single donor with or without the HLA-DRB1*03:01 allele is shown. (B) The frequencies of DR3-Ag85A or DR3-ApoB tetramer^+^ CD4^+^ T cells from 7 HLA-DRB1*03:01-bearing donors 7 days after MVA85A vaccination are shown. Each symbol represents an individual donor and bar represents the mean. (C) Representative flow cytometry plots of DR3-Ag85A tetramer staining of PBMCs collected before, and at the indicated time points, after MVA85A vaccination are shown from a single individual. (D) Longitudinal follow-up of DR3-Ag85A tetramer^+^ CD4^+^ T-cell frequencies in 7 HLA-DRB1*03:01-bearing donors before, and up to 1 year after, MVA85A vaccination is shown. (E) The frequencies of IFN-γ-expressing T cells in the same 7 HLA-DRB1*03:01-bearing donors, measured by ELISpot assay after stimulation of PBMCs with 15-mer peptides spanning the entire Ag85A protein are shown. (F) Representative flow cytometry plots of CD38 expression on Ag85A-specific CD4^+^ T cells before and 7 or 14 days after MVA85A vaccination are shown in an individual. Cells were gated on DR3-Ag85A tetramer^+^ CD4^+^ T cells. (G) Longitudinal postvaccination follow-up of Ag85A-specific CD4^+^ T-cell activation in the 7 HLA-DRB1*03:01-bearing donors is shown. Activation was measured as CD38 median fluorescence intensity on DR3-Ag85A tetramer^+^ CD4^+^ T cells. *p*-values were calculated using the Wilcoxon-matched pairs test.

Because the antigen-induced activation of T cells during in vitro stimulation may change the expression of certain phenotypic markers [14–16], we employed HLA class II tetramers to detect and characterize CD4^+^ T cells directly ex vivo, in the absence of T-cell activation. To establish whether CD4^+^ T-cell binding to the DR3-Ag85A HLA class II tetramer was specific, we thawed peripheral blood mononuclear cells (PBMCs) collected 7–14 days after MVA85A vaccination from seven individuals bearing the HLA-DRB1*03:01 allele. Cells were stained either with the DR3-Ag85A tetramer, or the DR3-ApoB control tetramer, which is complexed to a peptide spanning amino acids 2877–2894 from apolipoprotein B, a human protein involved in cholesterol transport [17]. DR3-Ag85A tetramer^+^ CD4^+^ T cells were detected in all seven vaccinees at frequencies between 0.015 and 0.53% (Fig. 1A). By contrast, DR3-ApoB tetramer^+^ CD4^+^ cells were detected at a median frequency of 0.017% (maximum frequency 0.024%) in these individuals (Fig. 1B). We also stained PBMCs from six HLA-DRB1*03:01 nonbearing MVA85A vaccinees, who had robust Ag85A-specific CD4^+^ T-cell responses observed previously by IFN-γ ELISpot assay (data not shown [18]). No specific DR3-Ag85A tetramer staining was observed in these samples; frequencies of tetramer^+^ CD4^+^ T cells were consistently observed below 0.02% (data not shown). These data highlight the specificity of the DR3-Ag85A HLA class II tetramer, both in terms of peptide antigen and HLA molecule.

### Ag85A-specific CD4^+^ T-cell response peaks 7 days after MVA85A vaccination

Previous MVA85A studies in humans have measured cytokine expressing cells to determine the magnitude and kinetics of the Ag85A-specific T-cell response after MVA85A vaccination [18, 19]. We stained PBMCs collected before, and at multiple time points up to 1 year after MVA85A vaccination with the DR3-Ag85A tetramer. Prevaccination frequencies of DR3-Ag85A-specific CD4^+^ T cells were mostly low (Fig. 1C). Following MVA85A vaccination, frequencies of DR3-Ag85A-specific CD4^+^ T cells increased markedly in all vaccinees (Fig. 1D). The response peaked 7 days postvaccination and had returned to prevaccination levels after 2 months (Fig. 1D). This kinetic profile was remarkably similar to that of specific CD4^+^ T-cell frequencies measured by IFN-γ ELISpot assay, following incubation of PBMCs with peptides spanning the entire Ag85A protein (Fig. 1E**)**. However, Ag85A-specific CD4^+^ T cells detected by ELISpot assay remained at higher frequencies than those observed prevaccination for the entire follow-up period (Fig. 1E), indicating greater sensitivity when T-cell responses to the entire Ag85A protein are measured, and/or possibly greater sensitivity of the ELISpot assay.

### CD4^+^ T-cell activation after MVA85A vaccination is short lived

To investigate the kinetics and duration of T-cell activation after vaccination, we measured expression of the activation marker CD38 on tetramer^+^ CD4^+^ T cells (Fig. 1F). T-cell activation increased markedly by 7 days postvaccination and was short lived, as CD38 expression levels returned to baseline levels in most vaccinees by 14 days (Fig. 1F and G). These low CD4^+^ T-cell activation levels persisted throughout the remaining follow-up period.

### Activated MVA85A-induced CD4^+^ T cells express a skin-homing phenotype

**Figure 2:**
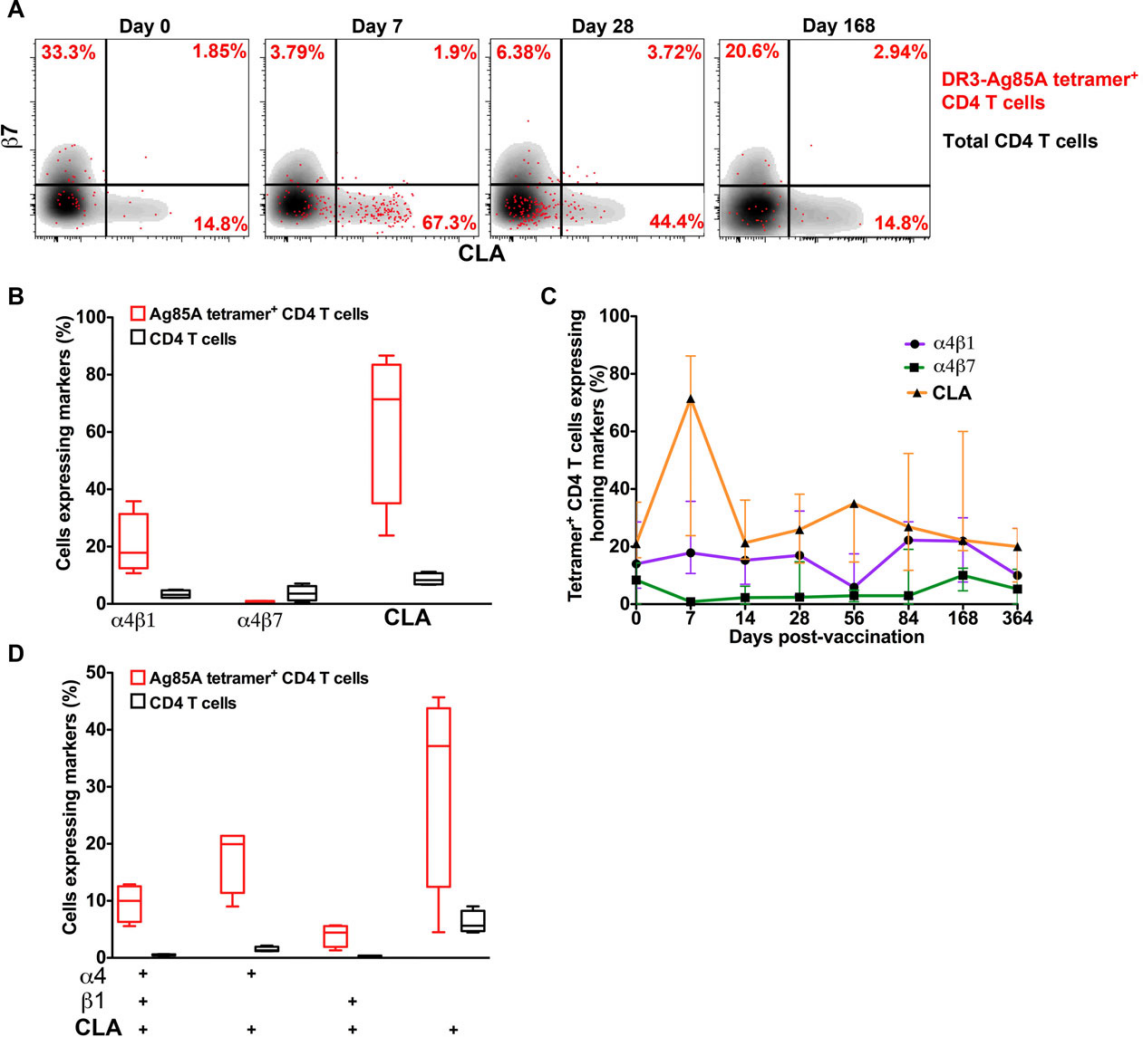
Homing marker expression by Ag85A-specific CD4^+^ T cells. (A) The expression of the T-cell homing markers CLA and integrin β7 on DR3-Ag85A tetramer^+^ CD4^+^ T cells (red dots) or the total CD4^+^ T-cell population (gray background) before or after MVA85A vaccination is shown for a single individual. Red numbers indicate proportions of DR3-Ag85A tetramer^+^ CD4^+^ T cells in each quadrant. (B) The expression of α4β1, α4β7, and CLA on DR3-Ag85A tetramer^+^ CD4^+^ T cells, and the total CD4^+^ T-cell population, in 7 HLA-DRB1*03:01-bearing individuals 7 days after MVA85A-vaccination is shown. Horizontal lines represent the medians; boxes the inter-quartile range (IQR) and whiskers represent the range. Representative flow cytometry plots of homing marker expression are shown in Supporting Information Fig. 1B. (C) Longitudinal homing marker expression by DR3-Ag85A tetramer^+^ CD4^+^ T cells in the 7 MVA85A recipients, before and up to 1 year after MVA85A vaccination. Lines represent medians and error bars represent the IQR. (D) Coexpression of α4β1, α4, or β1 by CLA^+^ DR3-Ag85A tetramer^+^ CD4^+^ T cells, in the 7 MVA85A recipients 7 days after MVA85A vaccination. Horizontal lines represent the medians; boxes the IQR and whiskers represent the range.

The capacity of antigen-specific T cells to traffic to the site of infection-induced inflammation is critical for protective immunity. To determine the tissue homing potential of Ag85A-specific CD4^+^ T cells induced by intradermal MVA85A vaccination, we measured expression of homing markers associated with trafficking to skin (cutaneous lymphocyte antigen, CLA [20]), gut (α4β7 [21]), and lung (α4β1 [22]) on DR3-Ag85A tetramer^+^ CD4^+^ T cells (Fig. 2A). During the peak response, 7 days postvaccination, Ag85A-specific CD4^+^ T cells predominantly expressed CLA, while a minority expressed α4β1 (Fig. 2B). This expression pattern was short lived and mirrored T-cell activation; by day 14 postvaccination the proportion of CLA-expressing cells had returned from ∼70% to prevaccination levels of ∼20%, and remained at this level throughout the duration of follow-up (Fig. 2C). The proportion of α4β1 expressing tetramer^+^ CD4^+^ T cells remained relatively consistent at ∼20% during follow-up. Ag85A-specific T cells expressing the gut homing marker, α4β7, were infrequent or not detectable, at all time points (Fig. 2C). Of note, more than 60% of the tetramer^+^ CD4^+^ T cells detected 14 days after vaccination expressed none of the homing markers analyzed.

An important observation was that expression of CLA, α4β1, and α4β7 was not distinct; many cells coexpressed these markers. Seven days postvaccination, CLA-expressing Ag85A-specific CD4^+^ T cells coexpressed the integrins α4β1, α4 alone or β1 alone (Fig. 2D). This coexpression pattern was not observed in the total CD4^+^ T-cell population.

### Activated MVA85A-induced CD4^+^ T cells display an effector phenotype

Vaccines that protect for decades, such as smallpox, induce a long-lived memory T-cell response [11, 23–25]. Such long-lived central memory (T_CM_) CD4^+^ cells, which home to lymph nodes by virtue of high CCR7 expression, produce mostly IL-2 and possess greater proliferative potential compared with effector (T_E_) or effector memory (T_EM_) CD4^+^ cells [26, 27]. The latter subsets migrate to sites of infection and predominantly express effector molecules, such as IFN-γ [26, 27].

**Figure 3:**
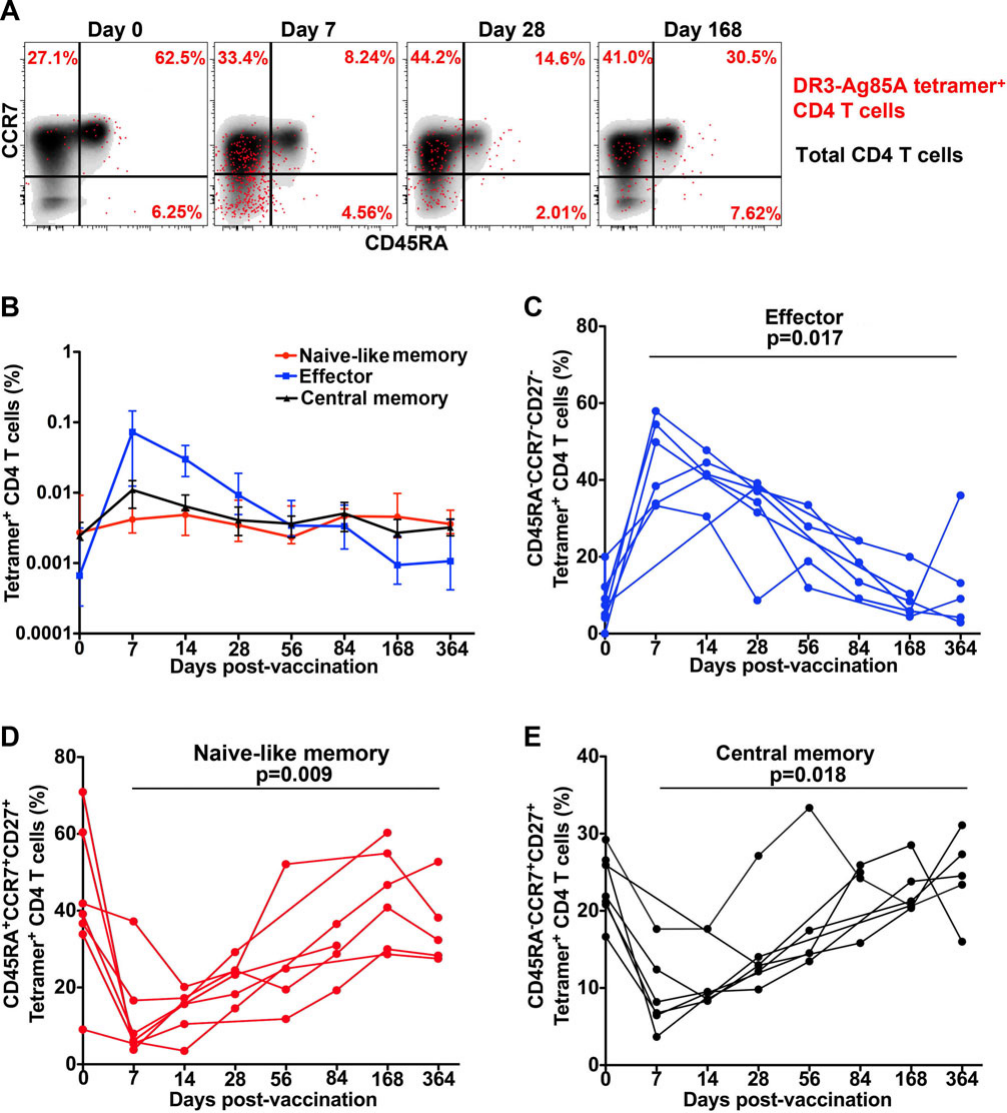
Memory phenotype of Ag85A-specific CD4^+^ T cells. (A) Longitudinal changes in expression of CD45RA and CCR7 by DR3-Ag85A tetramer^+^ CD4^+^ T cells (red dots) or the total CD4^+^ T-cell population (gray background) before or after MVA85A vaccination (*n* = 7). Red numbers indicate relative proportions of DR3-Ag85A tetramer^+^ CD4^+^ T cells in each quadrant. (B) Kinetic changes in frequencies of DR3-Ag85A tetramer^+^ CD4^+^ T cells expressing an effector phenotype (CD45RA^−^CCR7^−^CD27^−^), a “naive-like memory” phenotype (CD45RA^+^CCR7^+^CD27^+^) or a central memory phenotype (CD45RA^−^CCR7^+^CD27^+^) at the indicated time points after MVA85A vaccination. Data are shown as median + IQR of the seven donors. Representative flow cytometry plots of the gating strategy and memory marker expression are shown in Supporting Information Fig. 1C. (C–E) Kinetic changes in the proportions of DR3-Ag85A tetramer^+^ CD4^+^ T cells expressing (C) an effector phenotype (CD45RA^−^CCR7^−^CD27^−^), (D) a “naive-like memory” phenotype (CD45RA^+^CCR7^+^CD27^+^) or (E) a central memory phenotype (CD45RA^−^CCR7^+^CD27^+^) are shown. *p*-values were calculated using the Wilcoxon-matched pairs test.

To characterize the memory phenotype of MVA85A-induced T cells, we measured expression of CD45RA, CCR7, and CD27 on DR3-Ag85A tetramer^+^ CD4^+^ T cells (Fig. 3A). Ag85A-specific CD4^+^ T cells detected before MVA85A vaccination predominantly displayed either a CD45RA^+^CCR7^+^CD27^+^ phenotype, typical of naïve T cells [26, 27], and thus termed “naïve-like memory” T cells, or a CD45RA^−^CCR7^+^CD27^+^ T_CM_ phenotype (Fig. 3B and D). During the acute postvaccination response, when Ag85A-specific CD4^+^ T cells were highly activated (Fig. 1F), these cells predominantly displayed a CD45RA^−^CCR7^−^CD27^−^ effector phenotype (Fig. 3B and C). As this effector response waned, DR3-Ag85A tetramer^+^ CD4^+^ T cells reverted to displaying either the CD45RA^+^CCR7^+^CD27^+^ (Fig. 3B and D) or CD45RA^−^CCR7^+^CD27^+^ T_CM_ (Fig. 3B and E) phenotype, which predominated before vaccination.

### Increased proliferation and IL-2 expression of Ag85A-specific memory CD4^+^ T cells postvaccination

**Figure 4:**
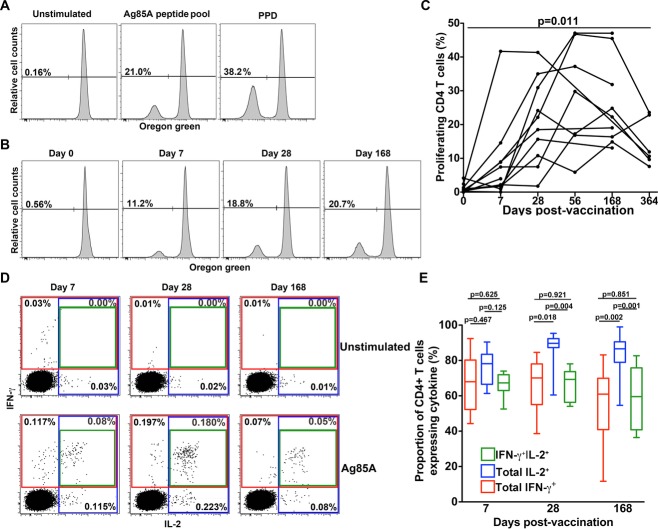
Lymphoproliferation of Ag85A-specific CD4^+^ T cells before and after MVA85A vaccination. PBMCs from MVA85A-vaccinated adolescents were stimulated with an Ag85A peptide pool, purified protein derivative or left unstimulated for 6 days. Proliferation was measured by dye dilution of Oregon Green. (A) Flow cytometry plots of specific CD4^+^ T-cell proliferation from a representative donor, 28 days after MVA85A vaccination are shown. Numbers indicate proportions of Oregon Green^low^, proliferating CD4^+^ T cells. (B) Ag85A-specific CD4^+^ T-cell proliferation before and after MVA85A vaccination, from a representative adolescent. (C) Longitudinal kinetics of Ag85A-specific CD4^+^ T-cell proliferation before and after MVA85A vaccination in ten adolescents are shown. (D) Representative plots of IFN-γ and IL-2 expression by unstimulated or Ag85A peptide pool stimulated CD4^+^ T cells, at the indicated time points after MVA85A vaccination. The red gate indicates IFN-γ-expressing CD4^+^ T cells, the blue gate indicates IL-2-expressing CD4^+^ T cells while the green gate indicates coexpression of both cytokines. Numbers indicate the frequencies of CD4^+^ T cells falling into these gates. (E) The relative proportions of total Ag85A-specific cytokine^+^ CD4^+^ T cells expressing total IFN-γ (red) or total IL-2 (blue) or both cytokines (green), at the indicated time points after MVA85A vaccination in 12 participants are shown as median, IQR (box) and range (whiskers). *p*-values were calculated using the Wilcoxon-matched pairs test.

To determine whether the phenotypes of pre-and postvaccination Ag85-specific CD4^+^ T cells were associated with differential T-cell proliferative capacity, we measured in vitro proliferation in response to Ag85A peptides before and up to 1 year after MVA85A vaccination (Fig. 4A and B). Prevaccination proliferation of Ag85A-specific CD4^+^ T cells was very low. Upon vaccination, Ag85A-specific in vitro proliferation of CD4^+^ T cells increased gradually and peaked between 28 and 168 days postvaccination. Frequencies of proliferating specific CD4^+^ T cells remained above prevaccination levels up to 12 months postvaccination (Fig. 4B and C).

CD4^+^ T cells that preferentially express IFN-γ generally have lower proliferative capacity, while predominant IL-2 expression is associated with greater proliferation [28, 29]. To further characterize the function of MVA85A-induced memory cells, we measured the relative proportions of Ag85A-specific CD4^+^ T cells expressing IFN-γ and/or IL-2 at 7, 28, and 168 days after MVA85A vaccination (Fig. 4D). Ag85A-specific CD4^+^ T cells at the prevaccination time point were too infrequent to analyze relative proportions of cytokine-expressing cells definitively (see methods). The acute response, 7 days postvaccination, was characterized by similar proportions of CD4^+^ T cells expressing IL-2 and/or IFN-γ. The waning of T_E_ cells after the peak response was associated with increasing proportions of Ag85A-specific IL-2-expressing cells and decreasing proportions of IFN-γ-expressing cells (Fig. 4D and E). However, most antigen-specific CD4^+^ T cells coexpressed IFN-γ and IL-2 (Fig. 4D).

### Naïve-like Ag85A-specific CD4^+^ T cells are not T memory stem cells

A novel, long-lived T-cell population, stem cell-like memory T (T_SCM_) cells, has recently been described in animals [30, 31] and humans [32]. These cells, which share phenotypic characteristics with CD45RA^+^CCR7^+^ naive T cells, possess an enhanced capacity for self-renewal and multipotent ability to derive T_CM_, T_EM_, and T_E_ cells [32].

**Figure 5:**
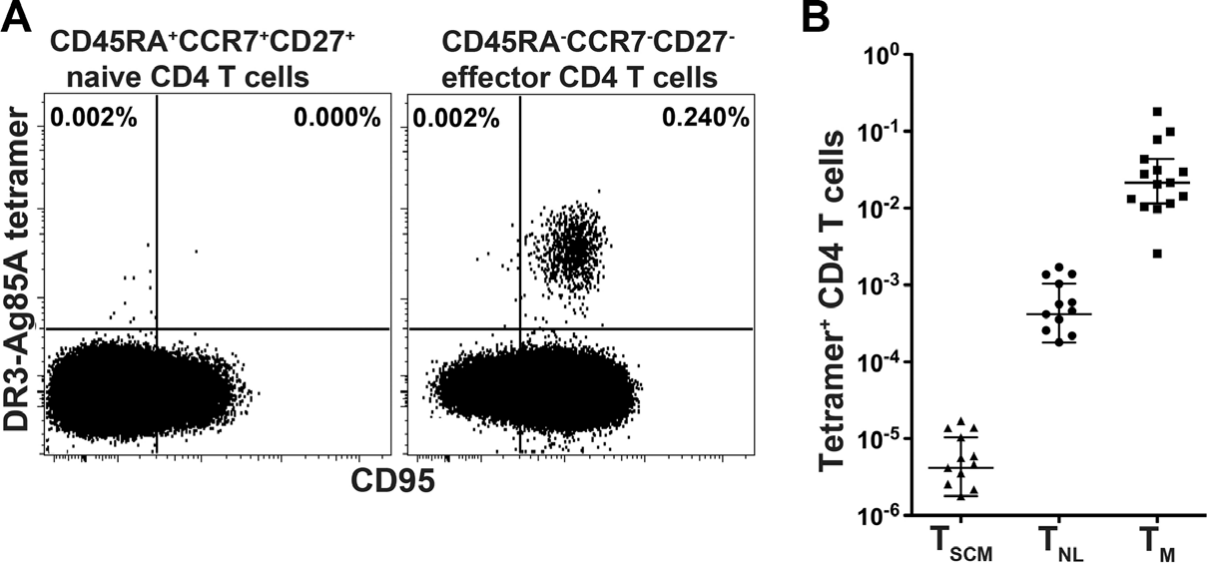
Phenotypic characterization of naïve-like Ag85A-specific CD4^+^ T cells. (A) Representative flow cytometry plots of CD95 and DR3-Ag85A tetramer staining of CD4^+^ T cells in PBMCs collected 14 days after MVA85A vaccination from an individual adolescent. Shown are cells gated on the total CD45RA^+^CCR7^+^CD27^+^ naïve CD4^+^ T cells (left) and cells gated on the total CD45RA^−^CCR7^−^CD27^−^ T_E_ CD4^+^ cells (right). The gating strategy implored to identify T_SCM_ cells is shown in Supporting Information Fig. 1D. (B) The frequencies of DR3-Ag85A tetramer^+^ CD4^+^ T cells classified as T_SCM_ cells, naïve-like memory (T_NL_) CD4^+^ T cells and CD45RA-memory (T_M_) CD4^+^ T cells in 4 HLA-DRB1*03:01-bearing participants, all postvaccination time points are shown. The values for three samples were 0, and are therefore not plotted on the logarithmic scale. Horizontal lines represent the medians, and error bars the IQR.

Since the frequencies of CD45RA^+^CCR7^+^CD27^+^ DR3-Ag85A tetramer^+^ CD4^+^ T cells were greater than those described for circulating pathogen-specific naïve T cells [33, 34], we hypothesized that they were T_SCM_. Because CD95 may discern T_SCM_ from naïve CD4^+^ T cells (Supporting Information Fig. 1D and [32]), we measured CD95 expression on DR3-Ag85A tetramer^+^ CD45RA^+^CCR7^+^CD27^+^ CD4^+^ T cells in PBMCs from adolescents who received MVA85A (Fig. 5A). Naïve-like Ag85A-specific CD4^+^ T cells were detected at frequencies 10–20-fold lower than Ag85A-specific CD45RA^−^ memory CD4^+^ T cells (Fig. 5B). In turn, CD95^+^ T_SCM_ comprised at most 5–10% of this naïve-like CD4^+^ T-cell subset, while being undetectable in some vaccinees (Fig. 5B). These data suggest that most of the CD45RA^+^CCR7^+^CD27^+^ naive-like memory CD4^+^ T cells are not T_SCM_ cells.

## Discussion

Here, we characterized the antigen-specific CD4^+^ T-cell response induced by MVA85A boost vaccination in adults, adolescents, and children from a TB endemic setting, where BCG is routinely administered at birth. It is not known which characteristics of the T-cell response may mediate superior protection against TB than those primed by BCG or natural infection [2, 3]. We proposed that a boost vaccine should induce long-lived modifications to the functional and/or phenotypic characteristics of preexisting mycobacteria-specific T cells.

We showed that Ag85A-specific CD4^+^ T cells predominantly coexpressed IFN-γ and IL-2, skin homing integrins and activation markers during the effector response, which was short lived. Contraction was marked by a transition to predominantly IL-2-expressing, CD45RA^−^CCR7^+^CD27^+^ or CD45RA^+^CCR7^+^CD27^+^ specific CD4^+^ T cells.

The proportion of Ag85A-specific CD4^+^ T cells bearing effector or central memory phenotypes was similar before and 1 year after vaccination, but functional differences could be shown: Ag85A-specific CD4^+^ T-cell proliferative capacity was markedly higher 6–12 months after MVA85A than before vaccination, highlighting discordance between proliferative function and memory phenotype.

The short duration of Ag85A-specific CD4^+^ T-cell activation observed during the acute response to MVA85A was not surprising. Given the replication-deficient nature of the MVA vector [35], the presence of antigen is likely to be very short lived. The kinetics of this effector response are consistent with those reported for vaccination with live, rapidly cleared smallpox, and yellow fever vaccines [24]. Predictably, the duration of CLA expression by these effector cells also reflected the short-lived nature and location of the inflammatory response, which typically resolves within 7 days of vaccination and presents as redness and swelling at the intradermal injection site [18, 19]. Only a small proportion of DR3-Ag85A-specific T cells expressed α4β1, while α4β7 expression was negligible. We found that the homing markers CLA, α4, and β1 were coexpressed on Ag85A-specific CD4^+^ T cells during the acute response. Most previous studies have reported distinct expression patterns of these markers, implying that specific T cells possess homing potential to a single tissue site only [20–22, 36]. One study reported a similar finding in mice and humans, showing transient coexpression of CLA, and α4β7 [37]. Whether cells coexpressing homing markers may home to multiple sites is possible, but not definitive. These observations suggest that expression of homing markers may be more complex than previously acknowledged, and that studies of T-cell homing should take coexpression of these makers into account, especially while inflammation is present at the site of infection or vaccination.

Waning of the MVA85A-induced effector response coincided with a transition to CD45RA^−^CCR7^+^CD27^+^ T_CM_ and CD45RA^+^CCR7^+^CD27^+^ naïve-like phenotypes, which also predominated the Ag85A CD4^+^ T-cell response before MVA85A vaccination.

The observed T_CM_ phenotype of Ag85A-specific CD4^+^ T cells following MVA85A vaccination contradicts our previous finding in adolescents, which showed that antigen-specific T cells predominantly displayed a T_E_-cell phenotype up to 2 months postvaccination [18]. This discrepancy is likely due to the different assays employed to detect Ag85A-specific T cells. In our previous study, Ag85A-specific T cells were identified as cytokine-expressing CD4^+^ T cells following 12 h of in vitro restimulation with Ag85A peptides [18]. Short term in vitro T-cell stimulation has been shown to alter expression of certain phenotypic markers [14, 16, 38], and may be a potential confounder in our peripheral blood measurements. By contrast, ex vivo detection of specific T cells by HLA tetramers offers more accurate measurement of T-cell phenotype, since it does not rely on T-cell activation. Regardless, our current data of Ag85A-specific T-cell phenotype, cytokine expression, and proliferative potential, following MVA85A vaccination support the well-described differences in function between T_E_ and T_CM_ cells [27–29].

Whether long-lived memory cells with excellent proliferative potential, rather than effector functions, may confer better protection against TB is not known. A gradual loss of BCG-induced T cells through attrition has been mooted as an underlying reason for the waning of BCG-induced protection against TB observed during adolescence [39]. Long-lived T_CM_ responses can provide protection for decades as illustrated by successful prophylactic vaccines, such as those against tetanus toxoid [40], yellow fever [24], and smallpox [24]. The high proliferative potential observed up to 1 year after MVA85A vaccination may thus reflect an ability to rapidly generate large numbers of specific effector cells upon infection, which may improve longevity of anti-mycobacterial immunity. Such longevity is further supported by our finding that elevated frequencies of Ag85A-specific CD4^+^ T cells persist up to 5 years after MVA85A vaccination, even in infants (Tameris et al., unpublished data). However, no evidence for efficacy against TB disease or *M. tb* infection was observed in infants after MVA85A vaccination in a recent phase IIb trial [10]. It is not known why MVA85A failed to confer protection over and above newborn BCG vaccination in this infant trial, or whether MVA85A would be more efficacious in the older populations studied here, who have greater frequencies of Ag85A-specific responses before and after MVA85A vaccination than infants [12]. The possible reasons underlying the observed lack of efficacy in infants, which may include route and/or age of administration, dose of the vaccine, the high rate of *M. tb* transmission in the trial population, or the magnitude, function and/or phenotype of the induced immune response, have been discussed in detail [10, 41].

Induction and maintenance of a persistent, specific T_EM_ response, by chronic antigen stimulation, has also been suggested as an effective strategy against chronic infections [42], including *M. tb*. The partially protective effect of BCG vaccination against *M. tb* challenge in mouse models may support this: BCG persists and replicates in mice [43] and thus maintains a consistent population of T_EM_ cells [44]. The reason for a more protective response may be the preferential homing of T_EM_ cells to peripheral sites of inflammation, such as the lung. This is supported by results from murine vaccination with a recombinant BCG vaccine that expresses the membrane-perforating listeriolysin and is devoid of the urease C gene [45]. This vaccine was shown to recruit more antigen-specific cells to the lung and enhance protection against *M. tb* than parental BCG. Regardless, studies are needed to determine which phenotypic and/or functional attributes of T-cell responses induced by BCG and novel vaccine candidates may be associated with long-lived protection in humans.

We decided to focus on vaccine-induced antigen-specific CD4^+^ T-cell responses because previous studies showed that MVA85A induced low or undetectable Ag85A-specific CD8^+^ T-cell responses [12, 18]. Other prime-boost strategies, such as those employing recombinant BCG or adenoviral Aeras402 [8, 46], did induce antigen-specific CD8^+^ T-cell responses.

Substantial proportions of mycobacteria-specific CD45RA^+^ CCR7^+^CD27^+^ or CD45RA^+^CCR7^+^ naïve-like CD4^+^ T cells have been reported in multiple studies [9, 47–49], but have not been characterized. BCG-specific naïve-like CD4^+^ T cells expressed cytokines in response to antigen stimulation [9, 47, 48] and were present at frequencies considerably greater than those described for pathogen-specific naïve T cells [33, 34]. A population of memory T cells expressing a naïve-like phenotype along with CD95 and displaying functional properties of stem cells has been described and termed T_SCM_ cells [32]. Here, we have shown that Ag85A-specific naïve-like CD4^+^ T cells were mostly CD95-negative, suggesting that these mycobacteria-specific cells are not T_SCM_ cells [32]. Our experiments on T_SCM_ cells were done on limited numbers of cryopreserved PBMCs from MVA85A-vaccinated subjects. Since T_SCM_ cells typically occur at very low frequencies in peripheral blood [32], we cannot definitively rule out that these cells exist in the mycobacteria-specific repertoire. In contrast, Ag85A-specific naïve-like CD4^+^ T cells were surprisingly abundant (similar in frequency to T_CM_). Additional studies are required to delineate the functional attributes of naïve-like CD4^+^ T cells and how they fit into the ontology of T-cell differentiation.

A limitation of our study was that our analyses were confined to T cells circulating in the peripheral blood. It is likely that, early after vaccination, most antigen-specific T cells traffic to the vaccination site and are thus not circulating in the periphery.

Another limitation of our approach was the use of a single tetramer complexed to a single Ag85A epitope. We cannot rule out that CD4^+^ T cells recognizing different Ag85A epitopes may yield different results to the ones reported here.

In conclusion, we report that a prime-boost vaccination strategy against TB in children, adolescents, and adults modulates the function of long-lived memory CD4^+^ cells and endow them with the capacity to proliferate readily upon secondary antigen encounter. Our recent phase IIb trial results suggest that these memory CD4^+^ cells may not be sufficient for protection against TB in infants [10]. More studies are needed to explore whether a greater magnitude, a qualitatively different, or a completely new immunological response is needed for protection against TB.

## Materials and methods

### Study participants, vaccination and follow-up, blood collection, and HLA typing

We accessed cryopreserved samples from a subset of participants (24 adults, 12 adolescents, and 24 children, Table1) who were enrolled into two previously completed phase I/IIa trials of MVA85A [18, 19]. Participants were all vaccinated with BCG at birth, were all HIV negative and had no evidence of *M. tb* infection, as defined by a negative ESAT-6/CFP-10 ELISPOT and a tuberculin skin test in duration of <15 mm, and all had a normal chest X-ray. Participants received a single intradermal dose of 5 × 10^7^ plaque-forming units of MVA85A over the deltoid region of the left arm, and were followed up for a minimum of 6 months [18, 19]. None converted to a positive ESAT-6/CFP-10 response during follow-up. DNA was extracted from PBMCs using the QIAamp Mini Blood kit, following the manufacturer's instructions (Qiagen). High resolution HLA class I and II genotypes were determined for each participant by PCR using sequence-specific primers. HLA allele ambiguities were resolved by allele-specific DNA sequencing.

**Table 1:** Details of trial participants

Donor number	Age at enrollment (years)	Gender	Ethnicity	MVA85A vaccine trial	Assays performed	HLA-DRB1 genotype
DN01–1051	21	F	Black African	TB008	Tetramera), Elispotb)	^*^03:01, ^*^11:01
DN01–1078	42	M	Caucasian	TB008	Tetramer, Elispot	^*^03:01, ^*^12:01
DN01–1117	49	M	Mixed Race	TB008	Tetramer, Elispot	^*^03:01, ^*^12:01
DN04–1002	2	M	Mixed race	TB014	Tetramer, Elispot	^*^03:01, ^*^04:03
DN04–1011	6	M	Mixed race	TB014	Tetramer, Elispot	^*^03:01, ^*^15:03
DN02–1002	13	M	Black	TB008	Tetramer, Elispot, Prolic), ICSd)	^*^03:01, ^*^13:02
DN02–1006	15	F	Mixed race	TB008	Tetramer, Elispot, Proli, ICS	^*^03:01, ^*^13:02
DN02–1001	13	F	Black African	TB008	Elispot, Proli, ICS	^*^03:02, ^*^15:03
DN02–1003	13	M	Black African	TB008	Elispot, Proli, ICS	^*^11:01, ^*^14:01
DN02–1005	14	M	Black African	TB008	Elispot, Proli, ICS	^*^03:02, ^*^12:01
DN02–1007	15	M	Black African	TB008	Elispot, Proli, ICS	^*^09:01, ^*^13:01
DN02–1009	14	F	Black African	TB008	Elispot, Proli, ICS	^*^04:05, ^*^13:01
DN02–1011	15	F	Black African	TB008	Elispot, Proli, ICS	^*^01:02, ^*^12:01
DN02–1017	15	F	Black African	TB008	Elispot, Proli, ICS	^*^15:01, ^*^15:03
DN02–1020	15	M	Mixed race	TB008	Elispot, Proli, ICS	^*^11:01, ^*^13:01
DN02–1023	14	F	Mixed race	TB008	Elispot, Proli, ICS	^*^03:02, ^*^03:02
DN02–1025	15	M	Black African	TB008	Elispot, Proli, ICS	^*^01:02, ^*^03:02

a) Ex vivo HLA class II tetramer staining and phenotyping.

b) Ex vivo IFN-γ ELISpot assay.

c) In vitro proliferation assay.

d) Whole blood intracellular cytokine staining assay.

### IFN-γ ELISpot assay

The frequency of IFN-γ-expressing cells was measured by ex vivo ELISpot assay. Briefly, antigens included pooled Ag85A peptides (2 μg/mL each) and purified protein derivative (20 μg/mL). Medium alone served as negative control and phytohemagglutinin (Sigma-Aldrich, 10 μg/mL) as positive control. Results were expressed as the number of spot forming cells per million PBMCs above the negative control.

### Lymphoproliferation assay

PBMCs were thawed in 12.5% AB serum/RPMI media containing DNAse (20 μg/mL), washed and rested overnight at 37°C with 5% CO_2_ in medium. PBMCs were then stained with 0.5 μg/mL of Cell Trace Oregon Green 488 (Molecular Probes, Invitrogen) per 1 × 10^7^ cells as previously described [50]. Stained cells were incubated either with medium alone (negative control), 66 pooled 15 mer peptides overlapping by 10 amino acids, spanning the mycobacterial Ag85A protein (1 μg/mL each, Peptide Protein Research Ltd.) or *M. tb* purified protein derivative (from Statens Serum Institute, used as positive control at 2 μg/mL) for 6 days at 37°C with 5% CO_2_. Cells were stained with LIVE/DEAD Fixable Violet Dead Cell Stain (ViViD, Molecular Probes, Invitrogen) as previously described [50] before monoclonal antibody staining with the following antibodies: CD3 QuantumDot605 (clone UCHT1) from Invitrogen and CD8 PerCP-Cy5.5 (SK-1) from BD Biosciences.

### Whole blood intracellular cytokine assay

Briefly, 1-mL heparinized whole blood was incubated immediately after collection with antigens in the presence of anti-CD28 and anti-CD49d (each at 0.5 μg/mL, BD Biosciences). Pooled Ag85A peptides (2 μg/mL per peptide) or viable BCG (Strain Danish 1331, Statens Serum Institute, 1.2 × 10^6^ CFU/mL) were used as antigens. No antigen was used as a negative control, and Staphylococcal enterotoxin B (5 μg/mL, Sigma-Aldrich) as a positive control. After 7 hours, Brefeldin A (10 μg/mL, Sigma-Aldrich) was added and samples were incubated for a further 5 hours. Erythrocytes were lysed and white cells fixed using FACSLysing Solution (BD Biosciences), before cryopreservation. Cells were thawed in batch, permeabilized with BD Perm/Wash buffer and stained with the following fluorescent antibodies: CD3-Pacific Blue (UCTH1), CD8-PerCPCy5.5 (SK1), IFN-γ-AlexaFluor700 (K3), IL-2-FITC (5344.11), all from BD Biosciences, and CD4-QuantumDot605 (SK3) from Invitrogen.

### HLA class II tetramers and staining

Custom ordered PE-conjugated iTag MHC class II tetramers (100 μg/mL) were obtained from Beckman Coulter. The HLA-DRB1*03:01 tetramers were complexed either to the mycobacterial Ag85A 20 mer peptide, VPSPSMGRDIKVQFQSGGAN (DR3-Ag85A), or the human apolipoprotein B-100 peptide, ISNQLTLDSNTKYFHKLN, (DR3-ApoB, control tetramer) [17, 51]. Cryopreserved PBMCs were thawed, washed, and stained with Violet or Aqua LIVE/DEAD Fixable Dead Cell Stain. Cells were stained with 2 μg/mL iTAg class II tetramer at 37°C for 1 h as previously optimized [52]. Tetramer-stained cells were washed and stained with surface marker antibodies for 20 min at 4°C, except for staining with anti-CCR7-APC, which was done separately at 37°C for 20 min, before the following monoclonal antibodies were added: CD3 AlexaFlour 700 (UCHT1), CD14 V450 (MΦP9), CD19 V450 (HIB19), CD38 PeCy7 (HB7), CD8 PerCP-Cy5.5 (SK1), all from BD Biosciences. CD45RA PerCP-Cy5.5 (HI1700), β7 eFlour650 (FIB504), from eBiosciences; CD4 QuantumDot605 (S3.5), CD3 QuantumDot605 (UCHT1) from Invitrogen; α4 AlexaFlour647 (44H6), and β1 PECy7 (4B7R) from AbD Serotec; CLA FITC (HECA452) and CD95-allophycocyanin (DX2), from Biolegend, and CCR7-allophycocyanin (150503) from R&D Systems. Finally, cells were washed and fixed in 1% paraformaldehyde in PBS.

### Flow cytometry analysis

Stained cells were immediately acquired on a LSR II flow cytometer (BD Biosciences), configured to detect 13 parameters. Flow cytometry data analysis was performed with FlowJo version 9.2 (TreeStar). Unstained cells and single-stained mouse κ beads were used as controls and to calculate compensations for every run. Cell doublets were excluded using forward scatter–area versus forward scatter–height parameters (Supporting Information Fig. 1A); acquisition time gating was applied to exclude data with inconsistent fluorescence and antibody aggregates were gated out using “keeper” gating. Boolean gating was employed to discern memory populations as shown in Supporting Information Fig. 1D.

### Statistical considerations

For the intracellular cytokine-staining assay, the cut-off for a positive CD4^+^ T-cell response was above 0.01%, after frequencies of cells in the unstimulated sample had been subtracted. Phenotypic data were included for analysis only for samples with specific tetramer^+^ CD4^+^ T-cell frequencies above 0.02% and absolute numbers of tetramer^+^ CD4^+^ T cells of ≥35 cells. For the IFN-γ ELISpot assay, the cut-off for a positive response was 17 spot forming cell per million PBMCs, after the frequency of cells in the unstimulated sample had been subtracted, as previously reported [18].

Statistical tests were performed using Prism v.5.0a (GraphPad). Paired and unpaired comparisons were done using the nonparametric Wilcoxon-matched pairs, or the Mann–Whitney *U* tests, respectively.

## Abbreviations

*M. tb*: *Mycobacterium tuberculosis*
TB: tuberculosis
T_SCM_: stem cell-like memory T cell
