# Supplementary material for: Heterologous vaccination against human tuberculosis modulates antigen-specific CD4+ T-cell function
Source: Eur J Immunol. 2013 Jul 8;43(9):2409–20. doi: 10.1002/eji.201343454 (PMC3816254; doi:10.1002/eji.201343454)

European Journal of Immunology

Supporting Information

for

DOI 10.1002/eji.201343454

One B. Dintwe, Cheryl L. Day, Erica Smit, Elisa Nemes, Clive Gray, Michele Tameris, Helen McShane, Hassan Mahomed, Willem A. Hanekom and Thomas J. Scriba

Heterologous vaccination against human tuberculosis modulates antigen-specific $CD4^{+}$ T-cell function

© 2013 The Authors. *European Journal of Immunology* published by Wiley-VCH Verlag GmbH & Co. KGaA Weinheim.

Supporting Information Figure

Supporting Information Figure 1. Flow cytometric analysis and gating strategy of CD4 T cells. (A) Representative density plots showing the gating strategy employed to identify live, $CD3^+$, small $CD4^+$ lymphocytes. Cell doublets were excluded using forward scatter-area (FSC-A) versus forward scatter-height (FSC-H) parameters, small lymphocytes were then selected before gating on $CD19^-$ and $CD14^-$ live ($ViViD^{low}$), $CD3^+$ T cells. Finally, $CD4^+$ T cells were selected. (B) Representative flow cytometry plots showing $CD4^+$ T cell co-expression of the homing markers β7, β1, α4 and CLA. (C) Representative flow cytometry plots showing $CD4^+$ T cell co-expression of the memory markers CD45RA, CCR7, CD27 and CD95. (D) Representative flow cytometry plots showing the boolean gating strategy used to identify T_{SCM}. Expression of CD45RA, CCR7, CD27 and CD95 on DR3-Ag85A $tetramer^+$ $CD4^+$ T cell subsets (coloured dots) overlayed on the total $CD4^+$ T cell population (grey background) are shown. We decided to use only CD95 to discern between T_{SCM} and naïve cells, because expression of CD95 is markedly higher on T_{SCM} cells relative to naïve cells. By contrast, expression of IL-7Rα and IL-2Rβ, which are used by Gattinoni et al., did not provide additional ability to discriminate between these cell subset [32].

Supporting Information Figure 1

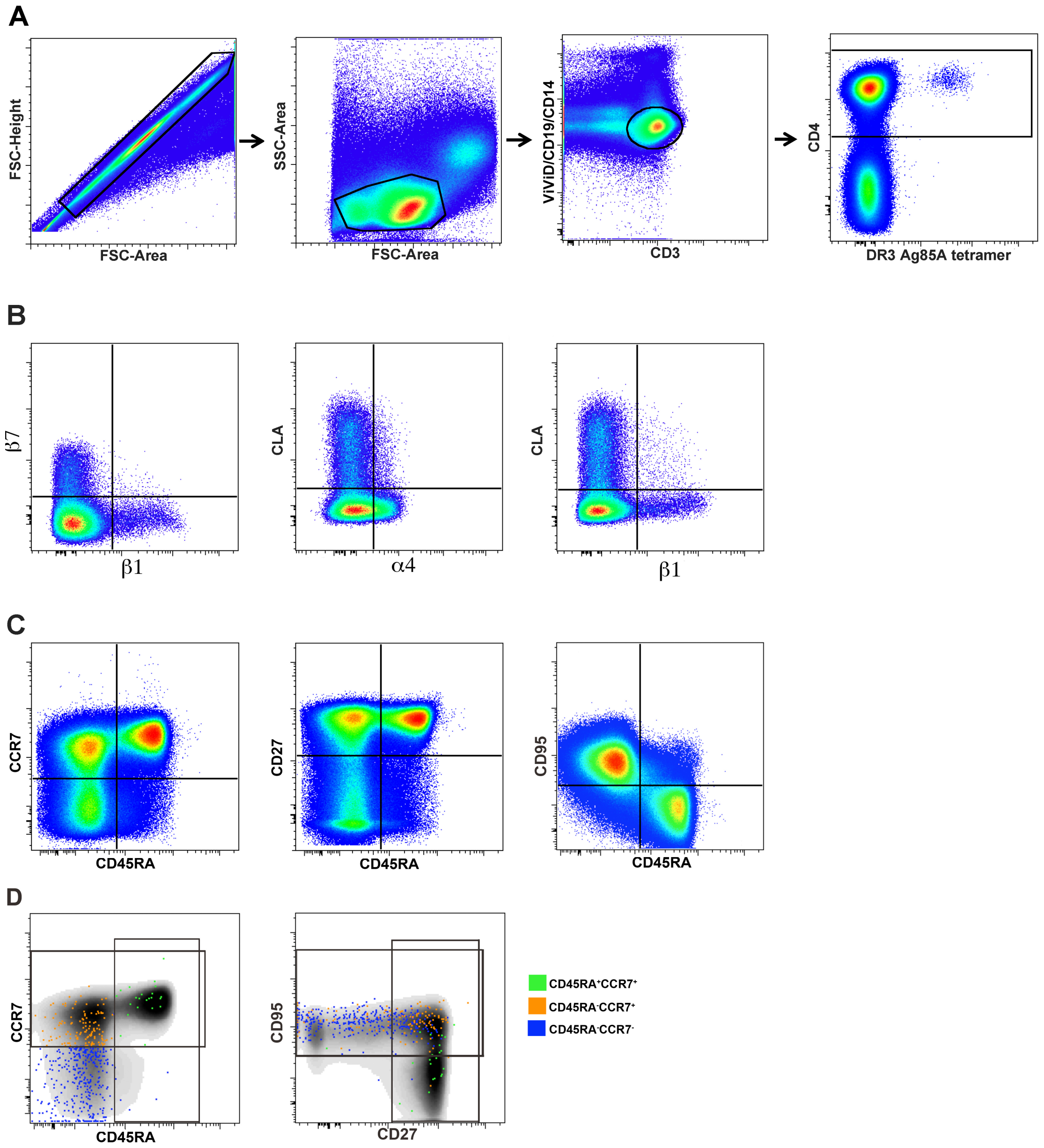

Supplement: Supplementary file 1 [file eji0043-2409-sd1.pdf]
